# Biomechanical Conditioning Enhanced Matrix Synthesis in Nucleus Pulposus Cells Cultured in Agarose Constructs with TGFβ 

**DOI:** 10.3390/jfb3010023

**Published:** 2012-01-05

**Authors:** Reshma K. Tilwani, Dan L. Bader, Tina T. Chowdhury

**Affiliations:** 1 School of Engineering and Materials Science, Queen Mary University of London, Mile End Road, London E1 4NS, UK; E-Mails: ex07009@qmul.ac.uk (R.K.T.); d.l.bader@qmul.ac.uk (D.L.B.); 2 School of Health Sciences, University of Southampton, Tremona Road, Southampton SO16 6YD, UK

**Keywords:** TGFβ, mechanical loading, matrix synthesis, nucleus pulposus, intervertebral disc

## Abstract

Biomechanical signals play an important role in normal disc metabolism and pathology. For instance, nucleus pulposus (NP) cells will regulate metabolic activities and maintain a balance between the anabolic and catabolic cascades. The former involves factors such as transforming growth factor-β (TGFβ) and mechanical stimuli, both of which are known to regulate matrix production through autocrine and paracrine mechanisms. The present study examined the combined effect of TGFβ and mechanical loading on anabolic activities in NP cells cultured in agarose constructs. Stimulation with TGFβ and dynamic compression reduced nitrite release and increased matrix synthesis and gene expression of aggrecan and collagen type II. The findings from this work has the potential for developing regenerative treatment strategies which could either slow down or stop the degenerative process and/or promote healing mechanisms in the intervertebral disc.

## 1. Introduction

Intervertebral disc degeneration commonly leads to chronic back pain and has reached epidemic proportions in the UK [1]. The main risks include mechanical, biochemical and genetic factors [2,3]. However, ageing and injury will inevitably contribute to the degenerative process [4]. The disease has enormous socioeconomic consequences with estimated costs in the UK exceeding £10 billion per year resulting from social and healthcare expenditure and loss of productivity in the workplace. Although clinical treatments are aimed at providing symptomatic pain relief, they do not restore biomechanical function or address disability. Accordingly, emphasis should be placed on developing regenerative treatment strategies which either slow down or stop the degenerative process and/or promote healing mechanisms. This information is essential for identifying the appropriate parameters suitable for pharmacological and physiotherapeutic interventions in disc regeneration. 

Previous *in vitro* and animal studies have utilized a range of growth factors to upregulate the extracellular matrix and reverse degenerative disc disease in bovine, human and murine disc tissues [5,6,7,8]. For example, there is evidence that insulin growth factor (IGF-1), transforming growth factor-β (TGFβ), bone morphogenetic proteins (BMPs) and growth differentiation factor-5 (GDF-5) regulate matrix production largely through autocrine and paracrine mechanisms [9,10,11,12]. Growth factors could therefore be used to enhance disc regeneration [8,13,14]. Furthermore, the anabolic response could be enhanced with the application of mechanical stimuli. Indeed, several research groups have developed bioreactor systems which enable application of physiological mechanical conditioning to cells cultured in 3D models. In particular, dynamic compression has been shown to maintain cellular phenotype and increased aggrecan gene expression and GAG content in human NP/alginate constructs [15]. This type of anabolic response was mediated by the integrins and has been reported to be dependent on the duration and type of compression regimen employed [15,16,17,18,19,20]. In contrast, static compression was found to have detrimental effects including reduced cell viability and matrix synthesis in IVD tissues [21,22,23,24]. The importance of these findings emphasizes the nature of the mechanical stimulus in controlling anabolic and catabolic activities in NP cells. Furthermore, the combined action of growth factors and mechanical loading in modulating matrix synthesis has not been previously examined. The present study therefore explored the specific effects of TGFβ and dynamic compression on NP cells cultured in agarose constructs. 

## 2. Experimental Section

### 2.1. NP Cell Isolation and Monolayer Culture

Bovine NP tissue (1.5 to 2.5 g) was isolated from caudal discs (3 discs per tail) of cattle aged less than 18 months from a local abattoir (Humphreys and Sons, Chelmsford, Essex, UK). Tails from 15 animals were used for all experiments. The tissue was diced and incubated on rollers in Dulbecco Modified Eagle Medium (DMEM)/F12 supplemented with penicillin/streptomycin (5%), ascorbic acid (2.5 µg/mL), amphoceterin B (5%), FCS (20%), collagenase type XI (0.8 mg/mL) and DNAse I (2.6 units/mL) for 16 h at 37 °C. The cell suspension was filtered through a 70 µM cell strainer, centrifuged at 2,000 rpm for 5 min and resuspended in media. Cells were subsequently expanded in monolayer culture in DMEM/F12 + 10% FCS until 80% confluent. 

### 2.2. Preparation of NP/Agarose Constructs

Confluent NP cells (Passage 1) were detached with 0.2% trypsin and 0.1% EDTA and counted on a hemocytometer using the trypan blue exclusion assay. Viable NP cells were seeded into 3% agarose type VII (low gelling temperature agarose) or 4% agarose type IX (ultra-low temperature gelling agarose) at 4 million cells/mL, using well-established methods, as previously described [25,26]. The agarose concentration and type were chosen based on previous observations [25,26]. To review briefly, the cells were resuspended in medium at a cell concentration of 8 × 10^6^ cells/mL and added to an equal volume of molten 6% (w/v) agarose type VII or 8% (w/v) agarose type IX in Earle Balanced Salt Solutions (EBSS) to yield a final cell concentration of 4 × 10^6^ cells/mL in 3% (w/v) agarose type VII or 4% agarose type XI (Sigma-Aldrich, Poole, UK). This is equivalent to approximately 400,000 cells per construct. The chondrocyte/agarose suspension was transferred into a sterile stainless steel mold, containing holes 5 mm in diameter and 5 mm in height and allowed to gel at 4 °C for 20 min. NP/agarose constructs were equilibrated in culture in DMEM/F12 + 10% FCS for up to 72 h to enable re-differentiation prior to further cell culture or mechanical loading experiments. The NP/agarose constructs were subsequently cultured for 48 h either under free-swelling conditions or when subjected to dynamic compression (15%, 1 Hz frequency). At the end of the culture period, constructs and media samples were stored at −20 °C prior to biochemical analysis or examined for mechanical testing. In addition, cell viability was assessed using calcein AM (5 µM) and ethidium homodimer (5 µM) by fluorescence microscopy (Invitrogen, Paisley, UK), as previously described [25].

### 2.3. Dose-Response Effect of TGFβ in NP Cells Cultured in Agarose Constructs

In separate experiments, equilibrated NP/agarose constructs (3%, type VII) were cultured for a further 48 h under free-swelling conditions in the presence and absence of 0.1, 1 and 10 ng/mL TGFβ_3_ (Cambrex Bioscience, Wokingham, Berkshire). At the end of the experiment, constructs and media samples were stored at −20 °C prior to biochemical analysis. 

### 2.4. Application of Dynamic Compression

In separate experiments, the combined effects of TGFβ and dynamic compression on protein synthesis and gene expression were examined in NP/agarose constructs (3%, type VII) cultured for 6 and 48 h. The bioreactor system has been extensively described previously [25,26]. To review briefly, equilibrated constructs were transferred into individual wells of a 24-well culture plate (Costar, High Wycombe, UK) and mounted within the bioreactor. One milliliter of media supplemented with either 0 or 10 ng mL^−1^ TGFβ were introduced into each well. Two separate compression regimens were applied to constructs in a dynamic manner at a strain amplitude of 15% and a frequency of 1 Hz:

▪ 1.5 h compression with a 22.5 h unstrained period repeated 2 × (1.5 h/22.5 h^×2^);
▪ 1.5 h compression with a 4.5 h unstrained period repeated 8 × (1.5 h/4.5 h^×8^).

Control constructs were maintained in an unstrained state within the bioreactor system and cultured for the same time period. At the end of the culture period, the constructs and corresponding media were immediately stored at −70 °C prior to analysis. 

### 2.5. RNA Isolation, cDNA Synthesis and Real-Time qPCR

RNA was isolated from NP cells cultured in agarose using protocols described in the QIAquick^®^ Spin gel extraction and RNeasy^®^ kits, as previously described (Qiagen, West Sussex, UK) [27]. Following manufacturer’s instructions, Ambion’s DNA-*free* DNase treatment and removal reagents were used to eliminate any contaminating DNA from the RNA sample (Ambion Applied Biosystems, Warrington, UK). RNA was quantified on the Nanodrop ND-1000 spectrophotometer (LabTech, East Sussex, UK) and reverse transcribed (200 ng) in a 20 µL reaction volume using the manufacturer-supplied oligo (dT) primers. Minus reverse transcriptase (NoRT) control reactions were prepared for each sample by omitting the Stratascript™ reverse transcriptase. Real-time quantitative PCR assays coupled with molecular beacon probes were performed in 25 µL reaction mixtures containing 1 µL cDNA, 12.5 µL Brilliant^®^ QRT-PCR Master Mix, primer pairs and probes listed in Table 1 and nuclease free PCR grade water to 25 µL (Sigma Genosys, Cambridge, UK). Each sample was run in duplicate on the 96-well thermal system of the M × 3000P quantitative PCR instrument (Stratagene, Amsterdam, the Netherland). 

**Table 1:** Description of the Beacon designer sequences used to quantify gene expression.

Gene	Accession number	Sequences
Aggrecan	U76615	*Probe:* 5'-FAM-CGCGATCCACTCAGCGAGTTGTCAGGTTCTGAGATCGCG-DABCYL-3'
		*Forward:* 5'-TGGTGTTTGTGACTCTGAGG-3'
		*Reverse:* 5'-GATGAAGTAGCAGGGGATGG-3'
Collagen type II	X02420	*Probe:* 5'-FAM-CGCGATGCGTCAGGTCAGGTCAGCCATATCGCG-DABCYL-3'
		*Forward:* 5'-AAACCCGAACCCAGAACC-3'
		*Reverse:* 5'-AAGTCCGAACTGTGAGAGG-3'
GAPDH	U85042	*Probe:* 5'-HEX-CGCGATCCACCATCTTCCAGGAGCGAGATCCGATCGCG-DABCYL-3'
		*Forward:* 5'-TTCAACGGCACAGTCAAGG-3'
		*Reverse:* 5'-TTCAACGGCACAGTCAAGG-3'

**Note:** The Beacon Designer software was used to design forward and reverse primer and probe sequences for molecular beacon applications (Sigma Genosys Ltd.: Cambridge, UK). Probes contain FAM or HEX as a 5'—reporter dye and DABCYL as 3'—quencher. Note that the arm sequences are underlined.

Thermocycling conditions comprised of an initial polymerase activation step at 95 °C for 3 min, followed by denaturation of 35 cycles at 95 °C for 30 s, annealing at 55 °C for 1 min and extension at 72 °C for 1 min. PCR efficiencies for optimal primer pair and probe concentrations were derived from standard curves (*n* = 3) by preparing a ten-fold serial dilution of cDNA from a sample which represented the untreated control at time zero conditions. The real-time PCR efficiencies (E) of amplification for each target was defined according to the relationship, E = 10^[−1/slope]^. The *R*^2^ value of the standard curve exceeded 0.9998 and revealed efficiency values between 0.99 and 1.01. 

Fluorescence data was collected during the annealing stage of amplification and data was analyzed on the MxPro™ qPCR software (version 3, Stratagene). Baselines and thresholds were automatically set by the RG-3000™ qPCR software and used after manual inspection. The cycle threshold (C_t_) value for each duplicate reaction was expressed as the mean value and the results were exported into Microsoft Excel for further analysis. Relative quantification of aggrecan and collagen type II signals were accomplished by normalizing each target to the reference gene, GAPDH and to the calibrator sample by a comparative C_t_ approach. For each sample, the ratio of target ∆Ct and reference ∆Ct was calculated, as previously described [27,28]. In addition, the C_t_ values for GAPDH remained stable with no changes detected under all culture conditions, suggesting its suitability as a reference gene.

### 2.6. Biochemical Analysis

The production of nitrite was determined in media samples and assayed spectrophotometrically at 540 nm using the Griess reaction. sGAG content was measured in media and constructs digested overnight at 37 °C with 10 U mL^−1^ agarase followed by 1 h at 60 °C with 2.8 U mL^−1^ papain (both Sigma Chemical Co., Poole, UK) using the DMMB assay [25,26]. Total DNA content which remained stable throughout the culture conditions was assayed using the Hoescht dye 33258 in agarase/papain digests.

### 2.7. Mechanical Properties

NP/agarose constructs were tested in unconfined compression with a mechanical test system (MTS Bionix 100, Billingshirt, UK) using stainless steel platens which were larger compared with the 5 mm diameter constructs. Samples were initially subjected to a 0.02 N tare load followed by a strain rate of 0.05% strain/s up to a maximum strain of 15%. This constant strain was maintained while the constructs relaxed until an equilibrium stress was reached. From the stress-strain response a tangent modulus was estimated, while an equilibrium modulus was calculated following stress relaxation [29]. 

### 2.8. Statistics

For free-swelling experiments, data represent the mean and SEM values of 12 replicates from three separate experiments. For the mechanical loading experiments, data represent the mean and SEM values of 15 replicates from four separate experiments. Statistical analysis was performed by a two-way analysis of variance (ANOVA) and the multiple *post hoc* Bonferroni-corrected *t*-tests to compare differences between the various treatment groups. In all cases, a level of 5% was considered statistically significant (*p* < 0.05).

## 3. Results and Discussion

Growth factors may contribute to disc regeneration by regulating cell-mediated remodeling events and matrix synthesis. Mechanical loading will additionally influence the cellular response and regulate downstream effects such as gene expression and protein biosynthesis. Bioreactors are therefore valuable tools for evaluating the effects of mechanical loading on signal transduction pathways in 3D culture systems. The present study therefore utilized the agarose/bioreactor model to examine its potential as an *in vitro* system for disc regeneration. This is the first study to examine the combined effect of TGFβ and dynamic compression on gene expression and protein synthesis in NP/agarose constructs. 

### 3.1. The effect of Agarose Concentration on NP Protein Synthesis and Resulting Mechanical Properties

**Figure 1:**
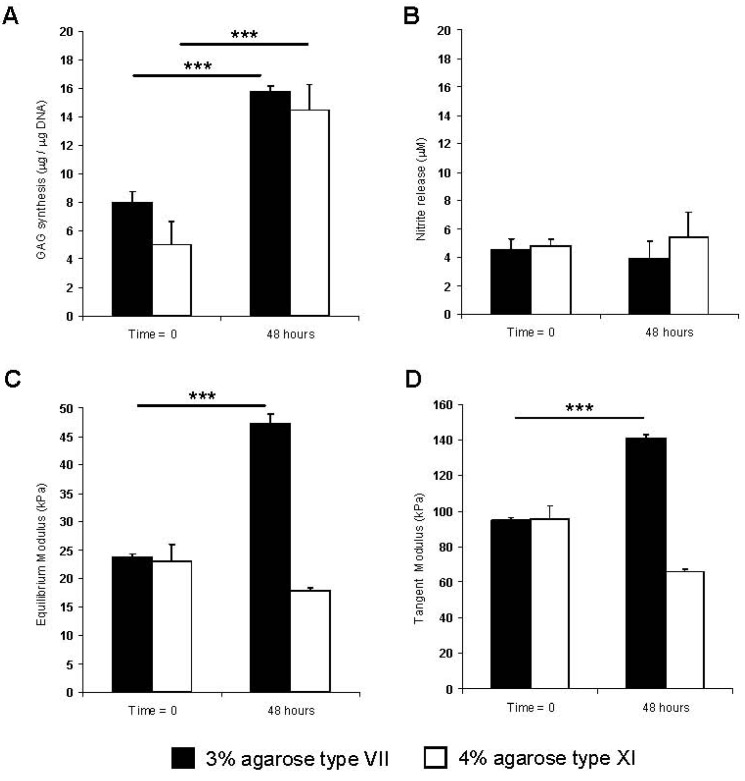
Effect of culture period on NP/agarose constructs. (**A**) GAG synthesis; (**B**) nitrite release; (**C**) equilibrium modulus (**D**) tangent modulus (*n* = 12). (***) indicates significant comparisons for the different treatment conditions (*p* < 0.001). All other comparisons were not significant (*p* > 0.05).

**Figure 2:**
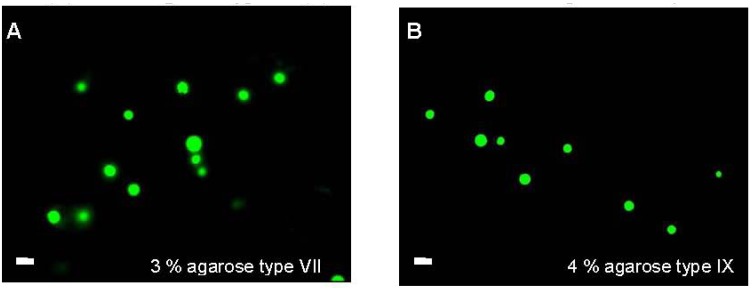
Confocal fluorescence images of viable NP cells cultured in 3% agarose type VII (**A**) and 4% agarose type IX (**B**) for 48 h. White bar represents 10 µm.

The effect of culturing NP cells in constructs of different agarose types and concentrations on protein synthesis and the resulting mechanical properties are illustrated in Figure 1. GAG synthesis was significantly enhanced over the 48 h culture period (*p* < 0.001) with minimal changes in nitrite release. The results for both moduli values revealed differences between the two constructs, with a significant increase only evident with 3% agarose type VII (*p* < 0.001). In addition, cell viability was maintained during the culture period, as shown by the confocal fluorescence images (Figure 2). The present data are in agreement with previous studies which utilized low-melting temperature agarose (1–3%) for maintaining NP anabolic activities [30,31]. In particular, long term culture of NP cells in agarose constructs increased cell proliferation and GAG synthesis and enhanced gene expression of aggrecan and collagen type II [32,33]. In addition, 3D cultures of NP cells were previously described in alginate beads or in fibrin/hylauronan and were shown to maintain cell phenotype and matrix synthesis [32,34,35,36]. Accordingly, subsequent experiments were limited to examining the effect of mechanical conditioning of NP cells cultured in 3% agarose type VII. 

### 3.2. The Effect of Dynamic Compression on Protein Synthesis in NP/Agarose Constructs

**Figure 3:**
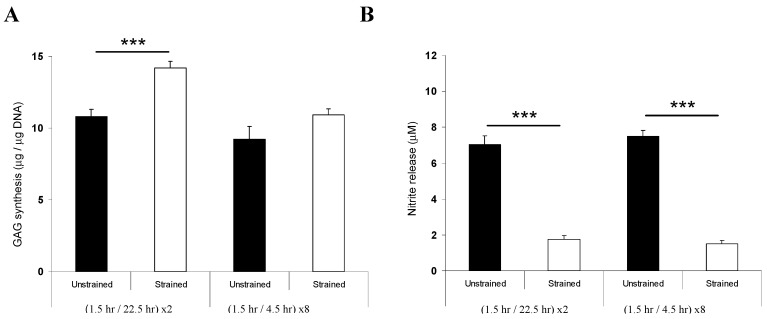
Effect of dynamic compression (15%, 1Hz) on protein synthesis in NP/agarose constructs (3%, type VII) cultured for 48 h.

The effect of two distinct dynamic compression regimens on NP protein synthesis is illustrated in Figure 3. In both cases, nitrite levels were significantly reduced by dynamic compression in NP/agarose constructs (*p* < 0.001). By contrast, the only significant upregulation in GAG synthesis was evident with the 1.5 h/22.5 h regimen (*p* < 0.001). GAG synthesis was therefore dependent on the temporal profile of mechanical loading, since a 1.5 h daily loading regimen favored GAG synthesis compared with a longer period of compression. This may therefore shift the balance of tissue remodeling in favor of catabolic over anabolic activity. Indeed, moderate levels of dynamic compression applied at low frequency (0.2–1 Hz) and magnitude (0.6–1 MPa) for short periods (2 h) have been shown to produce beneficial effects and increased aggrecan and collagen type II gene expression in rodent motion segments [16,37]. In addition, a single load for 30 min or daily for four weeks increased anabolic gene expression in human NP cells cultured in collagen gels [38,39]. Conversely, compression applied at high frequencies (>3 Hz) and magnitude (>1 MPa) for extended periods (>48 h) reduced matrix synthesis and increased gene expression of catabolic factors, such as nitric oxide (NO), matrix metalloproteinase-3 (MMP-3), matrix metalloproteinase-13 (MMP-13) and a disintegrin and metalloproteinase with thrombospondin motifs-4 (ADAMTS-4) [16,37]. The deleterious effects were shown to cause cell death and were similar to murine studies in which static compression was applied at high magnitudes (>1 MPa) for 6 hours [16,37]. 

In a separate study, stimulation by static compression for one week was associated with degenerative changes involving cell death, decreased aggrecan and collagen type II expression and elevated protease gene expression in murine disc tails [40,41,42,43]. Interestingly, these effects were not found at low magnitudes of static compression (<1 MPa) for short durations (<2 h) in bovine IVD [43]. Indeed, moderate levels of static compression (0.2–0.4 MPa) induced an anabolic response with increased proteoglycan and collagen synthesis and expression of collagen type II in explants or alginate constructs [43,44]. In general, dynamic compression which represents physiological stimuli promotes an anabolic response when compared to non-physiological loading which caused early signs of disc damage and catabolic remodeling, repair or degeneration [40,41,43,45,46]. Taken together, these findings suggest that low magnitudes of dynamic compression at a physiological frequency result in beneficial effects in disc tissue. However, results from previous *in vitro* studies are variable and will be dependent on the type of model system and compression regimen employed. Accordingly, the present study examined the effect of 1.5 h daily loading regimen, since this favored GAG synthesis and maintained basal levels of nitrite release in NP/agarose constructs cultured for 48 h. 

### 3.3. The Effect of TGFβ on Protein Synthesis in NP/Agarose Constructs

**Figure 4:**
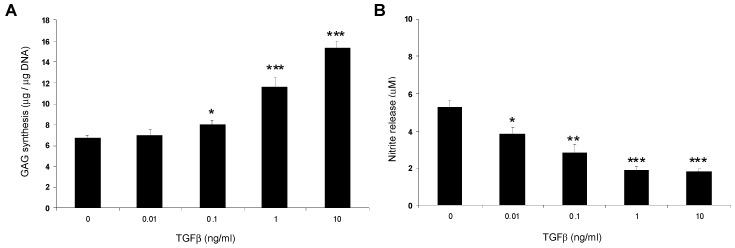
Dose-response effect of TGFβ (0.01 to 10 ng/mL) on GAG synthesis (**A**) and nitrite release (**B**) in NP/agarose constructs cultured for 48 h (*n* = 12). (*) indicates significant comparisons between untreated and TGFβ stimulated constructs.

The autocrine and paracrine production of growth factors is considered to be a major regulatory mechanism in IVD tissues [5]. We therefore examined the effect of TGFβ on protein synthesis in NP/agarose constructs. The presence of TGFβ significantly increased GAG synthesis and inhibits nitrite release in a dose-dependent manner (Figure 4). These data are in agreement with previous studies which showed dose-dependent upregulation of proteoglycan synthesis and gene expression of aggrecan and collagen type II by TGFβ in the agarose or alginate model [10,32]. At 1 ng/mL, TGFβ increased cell proliferation and proteoglycan synthesis in canine NP cells and the response was further increased with 20% FCS [8]. In rodent organ culture, TGFβ (5 ng/mL) maintained phenotype and proteoglycan synthesis when cultured in insulin-transferrin-selenium (ITS) containing media with 20% FCS [47]. At 10 ng/mL, NP cells cultured in collagen or hylauronan scaffolds with TGFβ results in the production of several types of proteoglycans and collagens [48]. However, matrix integrity could not be maintained during long term culture, highlighting the importance of choosing an optimal environment for TGFβ stimulation in 3D models. Furthermore, the expression of TGFβ and the TGFβ Type II receptor (TGFβRII) were found to be low in human herniated IVDs. In rat and murine models, TGFβ and its receptors were shown to decrease with age [49,50]. These findings support the potential of TGFβ in promoting matrix synthesis and maintaining tissue integrity and imply its absence may be a risk factor for IVD degeneration. 

### 3.4. The Effect of TGFβ and Dynamic Compression on Protein Synthesis and Gene Expression

**Figure 5:**
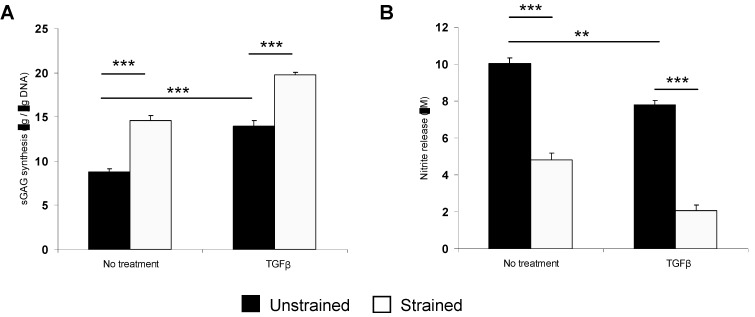
Effect of TGFβ and dynamic compression (15%, 1 Hz) on GAG synthesis (**A**) and nitrite release for 48 h (**B**). NP/agarose constructs were cultured with 0 or 10 ng/mL TGFβ for 48 h (*n* = 15). (*) indicates significant comparisons in unstrained and strained constructs as shown.

**Figure 6:**
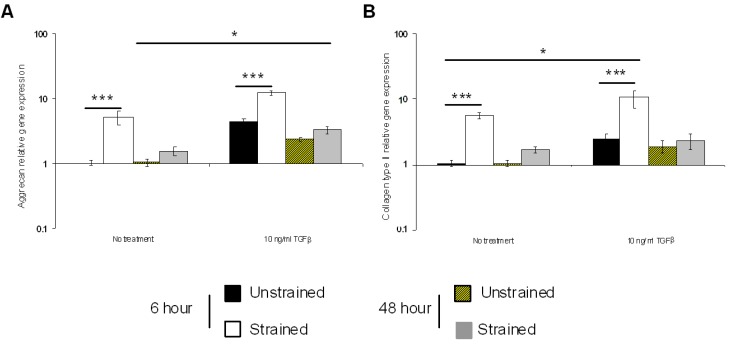
Effect of TGFβ and dynamic compression (15%, 1 Hz) on aggrecan (**A**) and collagen type II gene expression (**B**) for 6 and 48 h (B). NP/agarose constructs were cultured with 0 or 10 ng/mL TGFβ for 48 h (n = 6). (*) indicates significant comparisons in unstrained and strained constructs as shown.

The combined effect of TGFβ and dynamic compression on protein synthesis and gene expression in NP/agarose constructs are presented in Figure 5 and Figure 6, respectively. Stimulation by TGFβ and dynamic compression significantly enhanced GAG synthesis and reduced nitrite levels in NP/agarose constructs (both *p* < 0.001; Figure 5). In addition, TGFβ and dynamic compression significantly induced gene expression of aggrecan and collagen type II in a time-dependent manner (all *p* < 0.001; Figure 6). This is the first study to show an additive effect of TGFβ and dynamic compression on protein synthesis and gene expression in NP/agarose constructs. The combined effect of growth factors and mechanical loading on IVD metabolism is not known. Moreover, previous studies demonstrate enhanced chondrogenesis with TGFβ and dynamic compression resulting in anabolic gene expression in human mesenchymal progenitor cells cultured in monolayer or alginate [51,52,53]. It is possible that other growth factors such as GDF-5 or IGF-1 could initiate differentiation of MSCs to an IVD phenotype during physiological dynamic loading. However, the anabolic response will be dependent on the exposure time to growth factors and whether loading was applied during early or late stage cultures [54]. 

## 4. Conclusions

Growth factors and mechanical conditioning are important factors that determine cell behavior and tissue remodeling. This is the first study to show an additive effect of TGFβ and dynamic compression on protein synthesis and gene expression in NP/agarose constructs. Clinical application of TGFβ in combination with a pharmaceutical and physiotherapeutic approach could therefore be used to counteract the pathophysiological pathways for disc regeneration therapy. Further studies are needed to examine further the beneficial effects of mechanical loading and TGFβ on signal transduction pathways in maintaining normal IVD tissue remodeling.

## References

[1] Borenstein D.G. (2001). Epidemiology, etiology, diagnostic evaluation, and treatment of low back pain. Curr. Opin. Rheumatol..

[2] Pye S.R., Reid D.M., Smith R., Adams J.E., Nelson K., Silman A.J., O’Neill T.W. (2004). Radiographic features of lumbar disc degeneration and self-reported back pain. J. Rheumatol..

[3] MacGregor A.J., Andrew T., Sambrook P.N., Spector T.D. (2004). Structural, psychological, and genetic influences on low back and neck pain: A study of adult female twins. Arthritis Rheum..

[4] Adams M.A., Roughley P.J. (2006). What is intervertebral disc degeneration, and what causes it?. Spine.

[5] Osada R., Ohshima H., Ishihara H., Yudoh K., Sakai K., Matsui H., Tsuji H. (1996). Autocrine/paracrine mechanism of insulin-like growth factor-1 secretion, and the effect of insulin-like growth factor-1 on proteoglycan synthesis in bovine intervertebral discs. J. Orthop. Res..

[6] Wang H., Kroeber M., Hanke M., Ries R., Schmid C., Poller W., Richter W. (2004). Release of active and depot GDF-5 after adenovirus-mediated overexpression stimulates rabbit and human intervertebral disc cells. J. Mol. Med..

[7] Li X., Leo B.M., Beck G., Balian G., Anderson G.D. (2004). Collagen and proteoglycan abnormalities in the GD5-5 deficient mice and molecular changes when treating disk cells with recombinant growth factor. Spine.

[8] Thompson J.P., Oegema T.R., Bradford D.S. (1991). Stimulation of mature canine intervertebral disc by growth factors. Spine.

[9] Li J., Yoon S.T., Hutton W.C. (2004). Effect of bone morphogenetic protein-2 (BMP-2) on matrix production, other BMPs, and BMP receptors in rat intervertebral disc cells. J. Spinal Disord. Technol..

[10] Masuda K., Oegema T.J., An H.S. (2004). Growth factors and treatment of intervertebral disc degeneration. Spine.

[11] Le Maitre C.L., Richardson S.M., Baird P., Freemont A.J., Hoyland J.A. (2005). Expression of receptors for putative anabolic growth factors in human intervertebral disc: Implications for repair and regeneration of the disc. J. Pathol..

[12] Sai J.M., Hu Y.G., Wang D.C. (2007). Constructing adeno-associated virus-TGFbeta3 and comparing its biological effect on proteoglycan synthesis in dedifferentiated nucleus pulposus cells with adenovirus-TGFbeta1. Chin. Med. Sci. J..

[13] Le Maitre C.L., Hoyland J.A., Freemont A.J. (2004). Studies of human intervertebral disc cell function in a constrained *in vitro* tissue culture system. Spine.

[14] Cui M., Wan Y., Anderson D.G., Shen F.H., Leo B.M., Laurencin C.T., Balian G., Li X. (2008). Mouse growth and differentiation factor-5 protein and DNA therapy potentiates intervertebral disc cell aggregation and chondrogenic gene expression. Spine.

[15] Le Maitre C.L., Frain J., Millward-Sadler J., Fotheringham A.P., Freemont A.J., Hoyland J.A. (2009). Altered integrin mechanotransduction in human nucleus pulposus cells derived from degenerated discs. Arthritis Rheum..

[16] Maclean J.J., Lee C.R., Alini M., Iatridis J.C. (2004). Anabolic and catabolic mRNA levels of the intervertebral disc vary with the magnitude and frequency of *in vivo* dynamic compression. J. Orthop. Res..

[17] MacLean J.J., Lee C.R., Alini M., Iatridis J.C. (2005). The effects of short-term load duration on anabolic and catabolic gene expression in the rat tail intervertebral disc. J. Orthop. Res..

[18] Korecki C.L., Maclean J., Iatridis J.C. (2003). Dynamic compression effects on intervertebral disc mechanics and biology. Spine.

[19] Wang D.J., Jiang S.D., Dai L.Y. (2007). Biologic response of the intervertebral disc to static and dynamic compression *in vitro*. Spine.

[20] Walsh A.J., Lotz J.C. (2004). Biological response of the intervertebral disc to dynamic loading. J. Biomech..

[21] Ferguson S.J., Ito K., Nolte L.P. (2004). Fluid flow and convective transport of solutes within the intervertebral disc. J. Biomech..

[22] Gantenbein B., Grunhagen T., Lee C.R., van Donkelaar C.C., Alini M., Ito K. (2006). An *in vitro* organ culturing system for intervertebral disc explants with vertebral endplates: A feasibility study with ovine caudal discs. Spine.

[23] Haschtmann D., Stoyanov J.V., Ferguson S.J. (2006). Influence of diurnal hyperosmotic loading on the metabolism and matrix gene expression of a whole-organ intervertebral disc model. J. Orthop. Res..

[24] Korecki C.L., MacLean J.J., Iatridis J.C. (2007). Characterization of an *in vitro* intervertebral disc organ culture system. Eur. Spine J..

[25] Lee D.A., Knight M.M. (2004). Mechanical loading of chondrocytes embedded in 3D constructs: *In vitro* methods for assessment of morphological and metabolic response to compressive strain. Methods Mol. Med..

[26] Lee D.A., Bader D.L. (1997). Compressive strains at physiological frequencies influence the metabolism of chondrocytes seeded in agarose. J. Orthop. Res..

[27] Lee D.A., Brand J., Salter D., Akanji O.O., Chowdhury T.T. (2011). Quantification of mRNA using real-time PCR and Western blot analysis of MAPK events in chondrocyte/agarose constructs. Methods Mol. Biol..

[28] Pfaffl M.W., Tichopad A., Prgomet C., Neuvians T.P. (2004). Determination of stable housekeeping genes, differentially regulated target genes and sample integrity: BestKeeper Excel-based tool using pair-wise correlations. Biotechnol. Lett..

[29] Bader D.L., Knight M.M. (2008). Biomechanical analysis of structural deformation in living cells. Med. Biol. Eng. Comput..

[30] Walsh D.J., Bradford D.S., Lotz J.C. (2004). *In vivo* growth factor treatment of degenerated intervertebral discs. Spine.

[31] Shen B., Melrose J., Ghosh P., Taylor F. (2003). Induction of matrix metalloproteinase-2 and -3 activity in ovine nucleus pulposus cells grown in three-dimensional agarose gel culture by interleukin-1beta: A potential pathway of disc degeneration. Eur. Spine J..

[32] Gruber H.E., Fisher E.C., Desai B., Stasky A.A., Hoelscher G., Hanley E.N. (1997). Human intervertebral disc cells from the annulus: Three- dimensional culture in agarose or alginate and responsiveness to TGF-beta 1. Exp. Cell Res..

[33] Smith S.J., Chiaro J.A., Nerurkar N.L., Cortes D.H., Horava S.D., Hebela N.M., Mauck R.L., Dodge G.R., Elliot D.M. (2011). Nucleus pulposus cells synthesis a functional extracellular matrix and respond to inflammatory cytokine challenge following long-term agarose culture. Eur. Cell Mater..

[34] Chiba K., Andersson G.B., Masuda K., Thonar E.J. (1997). Metabolism of the extracellular matrix formed by intervertebral disc cells cultured in alginate. Spine.

[35] Maldonado B.A., Oegema T.R. (1992). Initial characterization of the metabolism of intervertebral disc cells encapsulated in microspheres. J. Orthop. Res..

[36] Melrose J., Smith S., Ghosh P. (2000). Differential expression of proteoglycan epitopes by ovine intervertebral disc cells. J. Anat..

[37] Walsh A.J., Lotz J.C. (2004). Biological response of the intervertebral disc to dynamic loading. J. Biomech..

[38] Neidlinger-Wilke C., Wurtz K., Liedert A., Schmidt C., Borm W., Ignatius A., Wilke H.J., Claes L. (2005). A three-dimensional collagen matrix as a suitable culture system for the comparison of cyclic strain and hydrostatic pressure effects on intervertebral disc cells. J. Neurosurg. Spine.

[39] Neidlinger-Wilke C., Liedert A., Wurtz K., Buser Z., Rinkler C., Kafer W., Ignatius A., Claes L., Roberts S., Johnson W.E. (2009). Mechanical stimulation alters pleiotrophin and aggrecan gene expression by human intervertebral disc cells and influences their capacity to stimulate endothelial migration. Spine.

[40] Lotz J.C., Colliou O.K., Chin J.R., Duncan N.A., Liebenberg E.B. (1998). Compression-induced degeneration of the intervertebral disc: An *in vivo* mouse model and finite-element study. Spine.

[41] Lotz J.C., Chin J.R. (2000). Intervertebral disc cell death is dependent on the magnitude and duration of spinal loading. Spine.

[42] Iatridis J.C., Mente P.L., Stokes I.A., Aronsson D.D., Alini M. (1999). Compression-induced changes in intervertebral disc properties in a rat tail model. Spine.

[43] Ohshima H., Urban J P., Bergel D.H. (1995). Effect of static load on matrix synthesis rates in the intervertebral disc measured *in vitro* by a new perfusion technique. J. Orthop. Res..

[44] Chen J., Setton L.A. (2004). Cell mechanics and mechanobiology in the intervertebral disc. Spine.

[45] Ariga K., Yonenobu K., Nakase T., Hosono N., Okuda S., Meng W., Tamura Y., Yoshikawa H. (2003). Mechanical stress-induced apoptosis of endplate chondrocytes in organ-cultued mouse intervertebral discs: An *ex vivo* study. Spine.

[46] Stokes I.A., Iatridis J.C. (2004). Mechanical conditions that accelerate intervertebral disc degeneration: Overload versus immobilization. Spine.

[47] Risbud M.V., Izzo M.W., Adams C.S., Arnold W.W., Hillibrand A.S., Vresilovic E.J., Vaccaro A.R., Albert T.J., Shapiro I.M. (2003). An organ culture system for the study of the nucleus pulposus: Description of the system and evaluation of the cells. Spine.

[48] Alini M., Li W., Markovic P., Aebi M., Spiro R.C., Roughley P.J. (2003). The potential and limitations of a cell-seeded/hyaluronan scaffold to engineer an intervertebral disc-like matrix. Spine.

[49] Okuda S., Nakasa T., Yonenobu K. (2000). Age-dependent expression of TGFβ1 and its receptors and age-related stimulatory effect of TGFβ1 on proteoglycan synthesis in rat intervertebral discs. J. Musc. Res..

[50] Matsunaga S., Nagano S., Onishi T., Morimoto N., Suzuki S., Komiya S. (2003). Age-related changes in expression of TGFβ and receptors in cells of intervertebral discs. J. Neurosurg..

[51] Angele P., Yoo J.U., Smith C., Mansour J., Jepsen K.J., Nerlich M., Johnstone B. (2003). Cyclic hydrostatic pressure enhances the chondrocyte phenotype of human mesenchymal progenitor cells differentiated *in vitro*. J. Orthop. Res..

[52] Miyanishi K., Trindale M.C., Lindsey D.P., Beeper G.S., Carter D.R., Goodman S.B., Schulman D.J., Smith R.L. (2006). Effects of hydrostatic pressure and TGFβ3 on adult human mesenchymal stem cell chondrogenesis *in vitro*. Tissue Eng..

[53] Campbell J.J., Lee D.A., Bader D.L. (2006). Dynamic compressive strain influences chondrogenic gene expression in human mesenchymal stem cells. Biorheology.

[54] Thorpe S.D., Buckley C.T., Venereal T., O’Brien F.J., Campbell V.A., Kelly D.J. (2008). Dynamic compression can inhibit chondrogenesis of mesenchymal stem cells. Biochem. Biophys. Res. Commun..

